# Real-Time Laser Interference Detection of Mechanical Targets Using a 4R Manipulator

**DOI:** 10.3390/s23052794

**Published:** 2023-03-03

**Authors:** Tingrui Liu, Zhongwei Ji, Yan Ding, Youfeng Zhu

**Affiliations:** College of Mechanical and Electronic Engineering, Shandong University of Science and Technology, Qingdao 266590, China

**Keywords:** mobile manipulator, interferometric sensing measurement, interferogram, spatial carrier, Fourier transform, spectrum filtering, extrapolation and interpolation

## Abstract

In this study, a laser interferometric sensing measurement (ISM) system based on a 4R manipulator system is developed to achieve detection of mechanical targets, which aims to realize the real-time, online detection of workpieces with high precision during processing. The 4R mobile manipulator (MM) system is flexible and can move in the workshop, aiming to preliminarily track the position of the workpiece to be measured and locate it at millimeter level. The reference plane of the ISM system is driven by piezoelectric ceramics with the spatial carrier frequency realized and the interferogram obtained by a charge coupled device (CCD) image sensor. The subsequent processing of the interferogram includes fast Fourier transform (FFT), spectrum filtering, phase demodulation, tilt elimination for wave-surface, etc., so as to further restore the surface shape of the measured surface and obtain the surface quality indexes. A novel cosine banded cylindrical (CBC) filter is used to improve the FFT processing accuracy, and a bidirectional extrapolation and interpolation (BEI) technique is proposed for the preprocessing operation of real-time interferograms before FFT processing. Compared with the results from a ZYGO interferometer, the real-time online detection results show the reliability and practicability of this design. The relative error of peak–valley value reflecting the processing accuracy can reach about 0.63%, with the root-mean-square value reaching about 1.36%. Some possible applications of this work include the surface of mechanical parts in the process of online machining, the end face of shaft-like structures, annular surfaces, etc.

## 1. Introduction

In recent decades, optical 3D measurement and shape reconstruction have been widely used in noncontact measurement applications, such as object recognition, medical practice, storage engineering, quality assurance, biometric safety, and unmanned transportation [1]. At present, popular 3D measurement and shape reconstruction methods include stereo vision, laser scanning, optical interferometry, photogrammetry, structured light technology, time-of-flight technology, etc. To date, all these technologies have made continuous progress in academic and business circles [2].

Structured light is one of the most popular technologies because it can simultaneously conduct high-speed and high-precision 3D shape measurement and plane reconstruction by using different fringe pattern processing methods [3]. For example, by considering the robustness of phase information to noise, ambient light, or reflectivity changes, the method of phase information based on a sinusoidal fringe pattern can achieve robust measurement. Among the phase-based methods, Fourier transform (FT) profilometry and phase shift (PST) profilometry are the most commonly used techniques for extracting phase information. A real-time 360-degree 3D surface defect detection method based on fringe projection profilometry (FPP) was proposed. This method does not need any auxiliary equipment for position control. It can identify the surface defects of complex objects in a very simple way in real time and accurately, saving a lot of operating costs for precision alignment and position orientation adjustment [4]. A real-time 3D shape measuring method based on dual-frequency spatial PST measuring profilometry was proposed, with two groups of spatial phase shifting deformed sub-patterns extracted from the corresponding deformed patterns to calculate the low-frequency and the high-frequency wrapped phases [5]. A high-speed, low-cost, and easy-to-implement 3D surface measurement approach for dynamic objects was demonstrated, with a dual-frequency dual-step PST extracting unwrapped phase by analyzing the influence of ambient light on the proposed method [6]. A new method combines fringe to phase network with FPP technology to achieve 3D reconstruction with excellent accuracy and speed performance, and finds a way to improve measurement performance based on supervised and unsupervised learning processes through depth learning [7]. System calibration is crucial to the FPP system because it determines how to convert the phase into 3D geometry. In the past few years, many calibration methods have been proposed. Some of the most commonly used calibration methods were reviewed, and the accuracy and implementation details were compared and summarized [8]. A novel dynamic deflectometry method with a simple system setup and calibration was also proposed for the 3D measurement of specular free form surfaces with high-speed and high-accuracy effects, which used a reference flat mirror to save calibration time and computer resources [9].

Laser interferometry is also a basic and common optical method in 3D measurement. A transient three-dimensional deformation and contour measurement method based on color electronic speckle interferometry (SPI) and wavelet transform was proposed. The wavelet transform algorithm was used to process each separated speckle interferogram, demodulate the deformation phase in the corresponding direction, and obtain the three-dimensional deformation of the measured object [10]. An integrated device based on SPI was developed to evaluate the 3D deformation of an infrared detector chip with temperature change, and a time PST based on parallel calculation of a general processor unit was proposed to realize quantitative real-time 3D measurement in three image layers [11]. The interferometric optical method integrates four-camera electronic SPI, and a new 3D shape reconstruction process was developed to measure the shape of the cornea and sclera shell and the whole-field mechanical deformation during an in vitro inflation test, with each camera providing accurate measurement of the laser beam phase related to the sample surface deformation [12]. Of course, various measurement methods are not limited to only one category. For example, speckle measurement is often constructed based on structured light and used for stereo vision analysis, and can obtain excellent measurement results. A novel randomly structured light system based on laser speckle pattern projection and its calibration procedure were proposed for applying computer vision methods to the measurement of vibration in featureless or reflective objects without modifying them, with vibration mode behavior and surface information obtained using a kind of full-field vibration measuring technique [13]. A preprocessing method in a stereo photogrammetry system based on histogram equalization and edge-preserving contrast enhancement is proposed to reduce subjective speckle noise. The amount of preprocessing varies with the distance of the object, thus optimizing 3D reconstruction. The results show that the density of 3D points is equivalent to that of professional 3D scanners with multiple lenses [14].

The realization of micro 3D surface detection technology based on laser interference technology has also been a research hotspot for a long time. With the development of intelligent manufacturing technology, the micro 3D surface detection technology of precision parts is an important research field [15]. The surface quality of precision parts requires not only comprehensive inspection after product processing, but also regular quality inspection during processing. In factory practice, in order to obtain a good surface characterization effect, in addition to relevant hardware facilities and high-precision algorithm processing, various measures such as industrial environment and real-time conditions should also be considered to achieve a dynamic detection process [16]. The laser interferometric dynamic measurement technology is used to process one-time interferograms, which can meet the requirements of high-precision measurement. The laser interferometric system based on the mobile manipulator frame can realize the tracking and positioning of the workshop site, and meet the requirements of real-time, online, dynamic, and continuous detection [17].

A mobile manipulator can complete some dangerous and special tasks by moving, and has practical value in many fields such as industry, national defense, etc. There are many kinds of mobile manipulators, the most common of which is the manipulator/robot moving on the ground by wheels. Taking the independent double rear-wheel differential-drive mobile manipulator as an example, it controls the speed and heading of the manipulator through the different speeds of the two rear wheels to achieve movement, track tracking, and positioning. Two models are commonly used, one is a kinematics model, which is used to solve the control problem between speed and position, and the other is a dynamics model, which is used to solve the control problem between speed and input force [18,19]. Its kinematics and dynamics analysis and control maturity are fully qualified for tracking and positioning in the workshop site.

The optical interferometers are used to measure various physical quantities in the measurement of small optical elements. The interesting information provided by the interferometer is the interferogram, which is a cosine phase function modulated by physical quantities (such as depth, displacement, and deformation) [20]. Existing case methods that are commonly used to extract the phase distribution of interferograms are the FT method, PST approach, and various improved algorithms of the two methods, as well as mutual fusion algorithms. FT technology processes the entire interferogram simultaneously, requiring only a one-shot image, but it requires a spatial carrier where pixels affect each other. By using the PST, each interferogram is processed separately, without affecting the other pixels. However, three, four, or more images are required. The space carrier phase shift (SCPS) method is a fusion algorithm which combines the advantages of the FT method and PST method. It can approximate the accuracy of the time domain phase shift method by processing a single space carrier interferogram [21], while the improved SCPS method can achieve the purpose of improving the accuracy through two or more interferograms [22].

Another improved SCPS algorithm based on the second-order difference was proposed by numerically calculating the first- and second-order difference of one spatial carrier frequency interferogram, and then high accuracy of phase extraction was obtained by using a normalization method [23]. The SCPS inherits the assumption in the traditional PST that all the unknown parameters are constant for the consecutive pixels involved in phase calculation. The SCPS is also a big basket of many PST algorithms from which a suitable one can always be selected for use. Kemao [24] introduced four typical algorithms in analysis and testing, which are the least squares algorithm, the synchronous detection algorithm, the windowed PST algorithm, and the Stoilov algorithm. Of course, SCPS can be used not only in interferograms but also in the measurement of projected fringe patterns (FPs). An excellent method was proposed to realize real-time 3D measurement with a radial SCPS algorithm characterized by the circular FPs continuously projected onto the moving object and the distorted FPs recorded by a CCD [25]. In the 3D reconstruction process, three FPs consisting of one recorded image and two artificial PST FPs were used to implement phase shifting, where the two artificial FPs were formed by numerically shifting the recorded image along the radial direction.

The FT method requires only a one-shot interferogram, which makes it the easiest method capable of dealing with dynamic situations, especially for real-time high-speed measurements. Due to the global characteristic of the FT for interferogram analysis, it is more tolerant to noise, and especially more effective for decreasing the influence of nonstationary noise [26]. Although the FT method can rapidly achieve the phase from a one-shot interferogram, its accuracy will be affected by the filtering window, the Gibbs effect, and carrier frequency [23]. To overcome the limitations of the FT method, time–frequency analysis techniques have been extensively developed over the past ten years, such as the window FT approach and the continuous wavelet transform approach [27]. To make the window FT approach more robust with a better anti-noise performance, it employed a short-time FT to analyze the interferogram locally by virtue of a window function, and used a ridge detecting method to determine the phase information. The wavelet transform approach is known as mathematical microscopy with flexible time–frequency analysis windows, which has been widely used in signal processing, and can recover corrupted data in interferograms. Hence, the FT algorithm has the advantage of high efficiency, thus making it still widely used in recent interferogram analysis. Based on the idea of making subtractions to the PST retrieved from a one-shot interferogram in the frequency domain directly to calculate the spectrum difference, an advanced FT method was proposed to mitigate the spectrum leakage problem due to the insufficient carrier, and reduce the edge errors caused by the Gibbs effect [28].

As mentioned earlier, the interferometric measurement method uses a CCD as the detector, which is actually a photoelectric sensor. It can obtain multi-point information of one surface at a time, and is one of the most important methods in optical precision measurement. The CCD photoelectric sensor mentioned above is used in the measurement of small optical elements, and has been widely used in micro-electro-mechanical systems and on-chip components, actuators, and electronic equipment, as well as photovoltaic thermal systems [29,30]. In fact, with the wide application of large plane optical elements in large astronomical telescopes and laser nuclear fusion systems, the performance of photoelectric sensors is also greatly improved. At the same time, a variety of interferometry-based methods continue to appear, such as the Ritchey–Common method, inclined incidence method, large-aperture interferometer, and sub-aperture splicing method [30]. Therefore, the performance of photoelectric sensors and optical instruments is also continuously improving.

An innovative design of photoelectric sensor based on porous silicon (PSi) was proposed to eliminate some problems of traditional PSi sensors, such as the undesired reflection caused by inaccurate positioning of optical fiber cable and PSi structure [31]. The optical response of devices based on PSi under different laser irradiation wavelengths in the range of 400–1100 nm has been deeply studied in order to observe the best optical response at a specific wavelength and obtain a high sensitivity [32]. The marginal change in geometric size caused by the change in external environment will affect the performance of optical instruments. Highly dimensionally stable materials can minimize these effects because they provide a low coefficient of thermal expansion. Therefore, Hamann et al. [33] proposed an improved interferometer that uses differential wavefront sensing to correct tilt to length coupling using angle measurement.

In addition, although the phase measurement interferometry technology has been widely reported, when the experimental conditions are not conducive to the experimenter’s implementation of phase shift, loading wave, and other modulation methods, the detector often has a single closed fringe. At this time, the commonly used phase shift demodulation technology and spectrum analysis methods are no longer applicable. The regularized phase following (RPF) technique can recover the phase of a single closed fringe, which is the most effective method for the phase recovery of a single closed fringe at present. In recent years, researchers have improved and developed RPF technology from the aspects of complex interferogram processing capability, algorithm stability, phase recovery accuracy, etc., gradually making it practical [34].

If the interferogram that is analyzed does not contain a tilt term (or not enough), then the complex intensities cannot be isolated and the FT method is not applicable as stated above. However, a bandpass filter of one-half of the spectrum is still possible. In such a case, the wrapped phase found is related to the actual one, solved by Maciel et al. [35]. Therefore, the FT method not only has the universality of application, but also has robustness of applicability.

**Figure 1:**
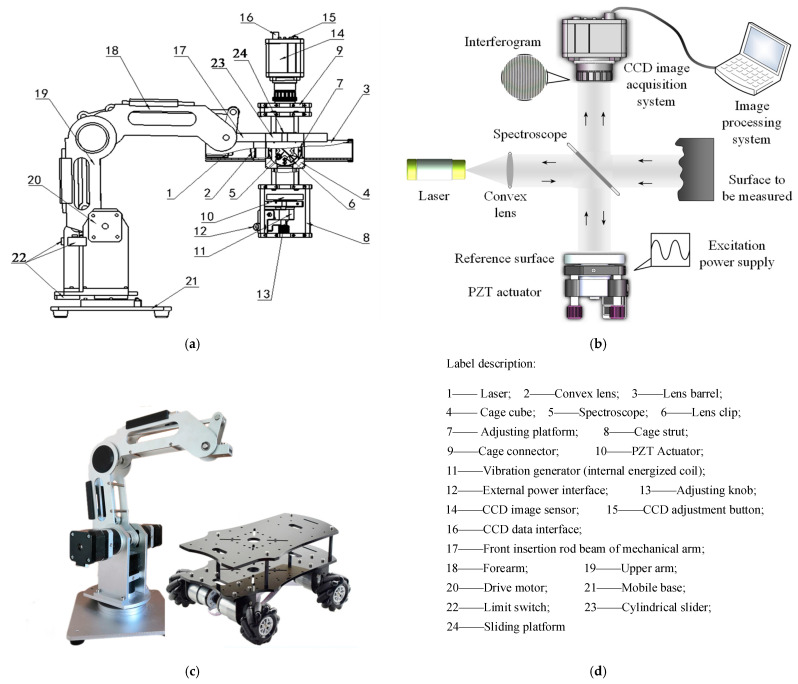
The whole measuring system and manipulator system: (**a**) the structural schematic diagram of laser ISM system and the 4R MM system; (**b**) the schematic diagram of laser interference; (**c**) the physical pictures of 4R MM system and mobile base; (**d**) the number label description.

In the present study, a real-time detection application scheme of a laser ISM system based on the 4R MM system is developed to realize online detection of precision micro-surfaces. The ‘4R’ here means four degrees of freedom of rotations, including the rotations at three joints and the rotational motion between the mechanical arm and the base (see Figure 1). The main contributions and characteristics of this study are summarized as follows. (1) The mobility of the MM system helps to realize real-time online detection of precision workpieces during workshop processing, which is different from the method of ‘unloading and transferring the workpiece to a special workshop for measurement’ in conventional detection; (2) the interferogram is processed by the FFT algorithm, and a cosine band cylindrical (CBC) filter is innovatively applied to improve the accuracy of spectral filtering; (3) bidirectional extrapolation and interpolation (BEI) technology is used to realize the squareness of circular and circular interferograms, and improve the accuracy of interferogram processing and the FFT algorithm; (4) for the measured surface in different ranges, it is necessary to select lasers with different wavelengths. In the test of the sample in this design, the optical response under different laser irradiation wavelengths in the range of 480–694 nm was tested. The optimum optical response was observed at the wavelength of 633 nm, and the corresponding sensitivity was determined as 9.4%. Therefore, the wavelength selected in this study is: λ=633 nm.

## 2. System Structure and Methods

### 2.1. System Structure

Figure 1 shows the concept, planning, and physical structure of the whole measuring system and manipulator system, including: (a) the structural schematic diagram of the laser ISM system and the 4R MM system. These two systems are designed independently of each other and connected together through front insertion rod beam 17 of the mechanical arm. The independence of the design facilitates the independent maintenance and upgrade of the laser ISM system and the 4R MM system, and is easy to install and disassemble; (b) the schematic diagram of the laser ISM system; (c) the physical pictures of the 4R MM system and mobile base; (d) number label description of the system structures 1–24.

The working principle of the laser interference system (b) is as follows. The light from the laser strikes the spectroscope and spreads in two ways. One path of light, reflected by the spectroscope, shines on the reference mirror, then returns to the original path, and enters the CCD camera through the spectroscope. The other path of light directly passes through the spectroscope, shoots to the surface of the object to be measured, returns after reflection, and enters the CCD camera after reflection by the spectroscope. The two beams of light interfere at the reception of the CCD camera to produce the interference FPs, which are transmitted to the PC through the image acquisition card and processed by the corresponding algorithms. The piezoelectric ceramic PZT actuator, using the inverse piezoelectric effect, generates high-frequency carrier signals under the excitation of external voltage signals.

As shown in Figure 1b, the measured surface is the vertical surface in the machining. In fact, this design is not limited to the detection of vertical surfaces. The vast majority of processed surfaces can theoretically be detected after a change in position or upgrading of the number of mechanical arms.

The main structures of the robot system include a 4R manipulator and mobile base, as shown in Figure 1c. The 4R manipulator has four degrees of freedom of rotations, including the rotations at three joints and the rotational motion between the mechanical arm and the base. The base is a movable four-wheel trolley with upper and lower double bottom plates. The manipulator is installed on the upper floor. The counterweight and controller hardware system, including power module, are installed between the upper and lower floors to prevent tipping.

The effectiveness and accuracy of robot system tracking, as well as the stability of system movement, are important indicators of robot system qualification. As front rod beam 17 and ISM system can be regarded as a whole, it is equivalent to an external load for the robot system. In the process of robot motion, there exists nonlinear vibration. We replaced the ISM system with a third-party load, and realized the tracking of the sinusoidal motion track in the process of motion. We investigated the stability of the robot motion process through iterative learning control, and studied the suppression method of nonlinear vibration interference [36].

In addition, the maintenance of the system is mainly reflected in the maintenance of the connectors of the mechanical system, because structural collision and looseness of the connectors will inevitably occur in the process of robot movement tracking and workpiece positioning. The reliability of the motion system is mainly guaranteed by the performance of the Siemens controller itself. With the upgrade of the controller hardware system, the reliability of the robot motion system will increase, and the performance will be further improved. Ji et al. [37] also realized the trajectory tracking and stability control of a robot system through double-loop sliding mode control, which verified the reliability of the tracking scheme. Further, with the rapid development of robot controller hardware, such as the application of various Siemens controller hardware, it is completely feasible to realize the motion planning in this design in the present study. Since various control algorithms of robot ‘stability analysis and trajectory tracking’ are more and more mature and shaped [18,19], this study ignores this aspect of analysis.

### 2.2. Methodology

#### 2.2.1. FT-Based Space Carrier Method

Using the inclined reference wavefront, the spatial carrier can be introduced into the interferogram, and its image can be represented as [26]:

(1) $$i\left(x,\;y\right)=a\left(x,\;y\right)+b\left(x,y\right)\cos[\varphi \left(x,\;y\right)+2\pi f_{0x}]$$

where a is the background intensity, b is the fringe amplitude, $f_{0}$ is the introduced spatial carrier frequency in the horizontal direction (*x* direction), and φ is the modulating phase. Further, rewrite Formula (1) as:

(2) $$i\left(x,\;y\right)=a\left(x,\;y\right)+c\left(x,\;y\right)\exp\left(2\pi jf_{0x}^{}x+2\pi jf_{0y}^{}y\right)+c^{*}\left(x,\;y\right)\exp\left(-2\pi jf_{0x}^{}x-2\pi jf_{0y}^{}y\right)$$

were $c\left(x,\;y\right)=\frac{1}{2}b\left(x,\;y\right)\exp\left[j\varphi \left(x,\;y\right)\right],$

It can be obtained by performing two-dimensional FT on Formula (2):

(3) $$I\left(u,\;v\right)=A\left(u,\;v\right)+C\left(u-f_{0x}^{},v-f_{0y}^{}\right)+C^{*}\left(u+f_{0x}^{},v+f_{0y}^{}\right)$$

Filter out the spectrum of the first harmonic with a filter and move it to the origin to obtain $C\left(u,\;v\right)$. $c\left(x,\;y\right)$ is obtained by inverse FT. The modulation phase can be described as:

(4) φx, y=tan−1Imcx,yRecx,y

#### 2.2.2. Filtering Based on CBC Filter

As mentioned above, the filtering process is indispensable. In the present study, a kind of CBC filter is innovatively applied to improve the accuracy of spectrum filtering processing and FT processing. The CBC filter is expressed as:

(5) $$h\left(x,\;y\right)=\left\{\begin{array}{cc} 0 & d_{f}>d_{\mathrm{fmax}}\\ 0.5\left[1+\cos\left(\pi \frac{d_{f}-d_{\mathrm{fmin}}}{d_{\mathrm{fmax}}-d_{\mathrm{fmin}}}\right)\right] & d_{\mathrm{fmax}}\geq d_{f}\geq d_{\mathrm{fmin}}\\ 1 & d_{f}<d_{\mathrm{fmin}} \end{array}\right.$$

where $d_{f}=\sqrt{{\left(x-h_{x}\right)}^{2}+{\left(y-h_{y}\right)}^{2}}$, $d_{\mathrm{fmax}}=L$, $d_{\mathrm{fmin}}=4L/5$. Herein, ($h_{x}$, $h_{y}$) is the central coordinate of the first harmonic spectrum; L is the distance between the fundamental frequency spectrum and the zero-frequency (i.e., the background frequency) spectrum.

In order to test the superiority of the CBC filter, a micro-paraboloid was simulated, with the carrier frequency added, and the FFT process carried out. By comparing the application performance of the rectangular filter and CBC filter, the performance superiority of the CBC filter was determined from the processing error of the wavefront (i.e., the wave-surface difference diagram). There are two factors for calibrating the wavefront error, i.e., the surface quality indexes: the peak and valley value (PVV) of the wave-surface and the root-mean-square value (RMSV) of the wave-surface [38]. 

**Figure 2:**
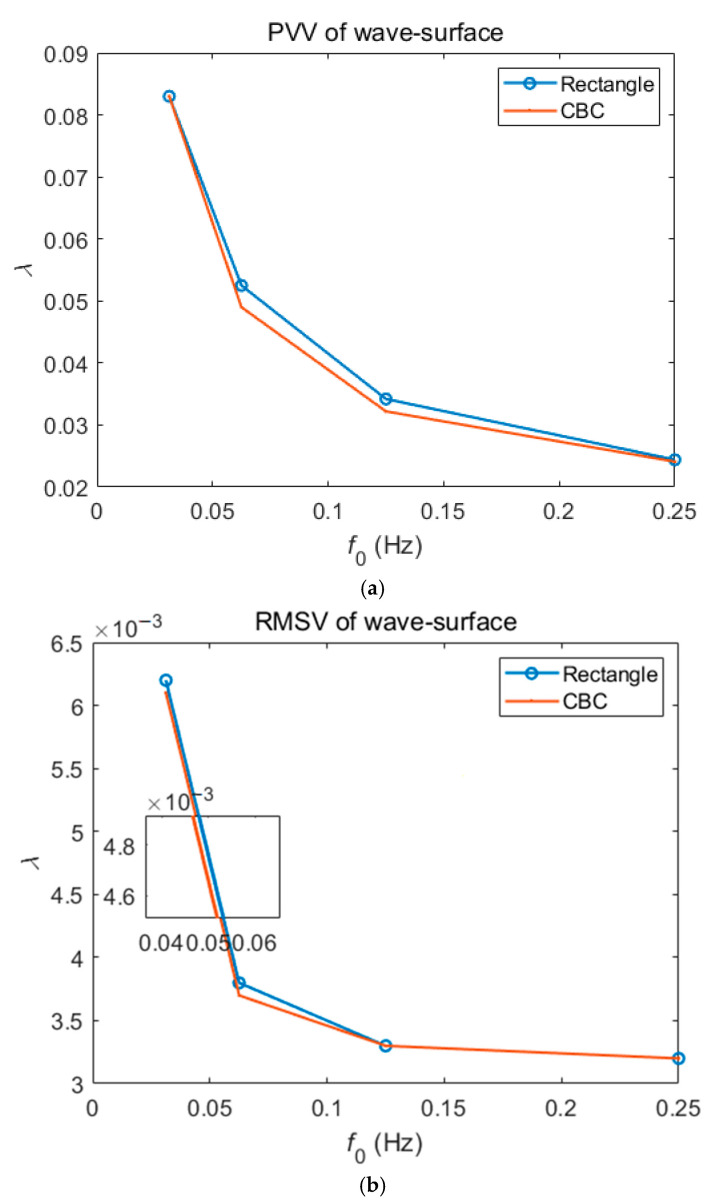
The PVVs and RMSVs under different carrier frequencies, $f_{0}=8/256,\;16/256,\;32/256,\;64/256\;\mathrm{Hz}$: (**a**) the PVVs based on different filters; (**b**) the RMSVs based on different filters.

In order to focus on the important part of the simulation results of this simulated wavefront, the PVVs and RMSVs were first shown under different carrier frequencies, $f_{0}=8/256,\;16/256,\;32/256,\;64/256\;$Hz, respectively, as demonstrated in Figure 2. The different results under two filtering operations by the rectangular filter and CBC filter are vividly demonstrated, and also detailed in Table 1. Note that the numerator of carrier frequency is the N-th power of 2, which is due to the inherent requirement of its fast FT algorithm in FFT processing. Generally speaking, too low carrier frequency means too low accuracy, and with the increase in carrier frequency, the accuracy will improve. However, too large carrier frequency not only represents the complexity of the carrier process and the reliability and hidden danger of the excitation power supply, but also makes the filtering operation impossible to complete after the carrier frequency increases to a certain value, such as the problems revealed later in the present study. Therefore, the operation at one of the medium carrier frequencies, $f_{0}=32/256$ Hz, was chosen as the research object. At the same time, the RSMVs in Figure 2b are relatively close, reflecting the robustness of the FT processing algorithm, while in Figure 2a, the results of the CBC filter at medium carrier frequencies reflect significantly higher accuracy. This is also the reason why this design in the subsequent actual measurement uses the CBC filter instead of the rectangular filter.

**Figure 3:**
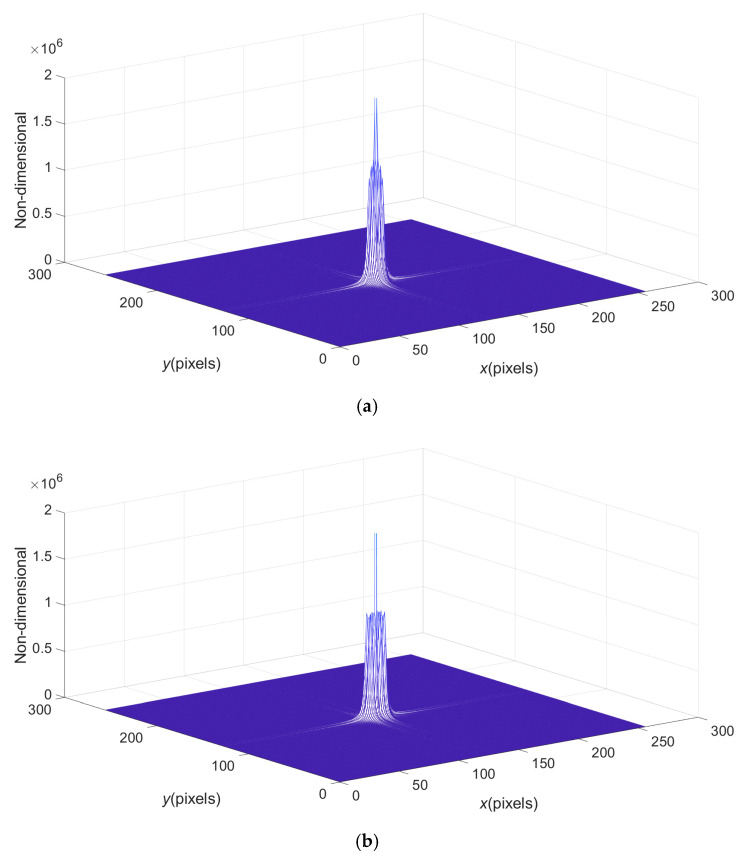
The spectrum diagrams that cannot be separated: (**a**) the carrier frequency is $f_{0}=2/256;$ (**b**) the carrier frequency is $f_{0}=4/256$.

In Table 1, an interesting fact is that under the action of too small carrier frequency, such as $f_{0}=2/256$ or $f_{0}=4/256$, the PVV after the operation of the rectangular filter is better than that after the operation of the CBC filter. This is because the carrier frequency is too small to separate the frequency spectrum of the fundamental frequency (the first harmonic) and the background spectrum, so the frequency aliasing state appears, as shown in Figure 3. Figure 3 shows the spectrum diagrams when carrier frequency is $f_{0}=2/256$, $f_{0}=4/256,$ respectively. In the case of frequency aliasing, the lower radius of the CBC filter (described in detail later) is larger than the upper radius, so the filtering error is just increased. In fact, due to the existence of frequency aliasing, the operation of these two kinds of filters has no practical significance, so in the present study, the low carrier frequencies are not considered.

#### 2.2.3. Simulation

A microscopic paraboloid is simulated as the measured surface. Assume that the function of optical path difference (*OPD*) of the two interference light waves is:

(6) $$OPD\left(x,y\right)=-[{\left(x-129\right)}^{2}+{\left(y-129\right)}^{2}]/16384\left(\lambda \right)$$

then the phase of the simulated wave-surface can be represented by *OPD*/2 $\left(\lambda \right)$.

The intensity $i_{0}$ of the original interferogram and the intensity i of the interferogram after carrier processing can be expressed as:

(7) $$i_{0}\left(x,y\right)=a_{0}+b_{0}\cos\left(2\pi OPD\right),\;i\left(x,y\right)=a_{0}+b_{0}\cos\left[2\pi \left(OPD+32/256\right)\right]$$

where $a_{0}=127$ and $b_{0}=200$ are background and contrast, respectively.

**Figure 4:**
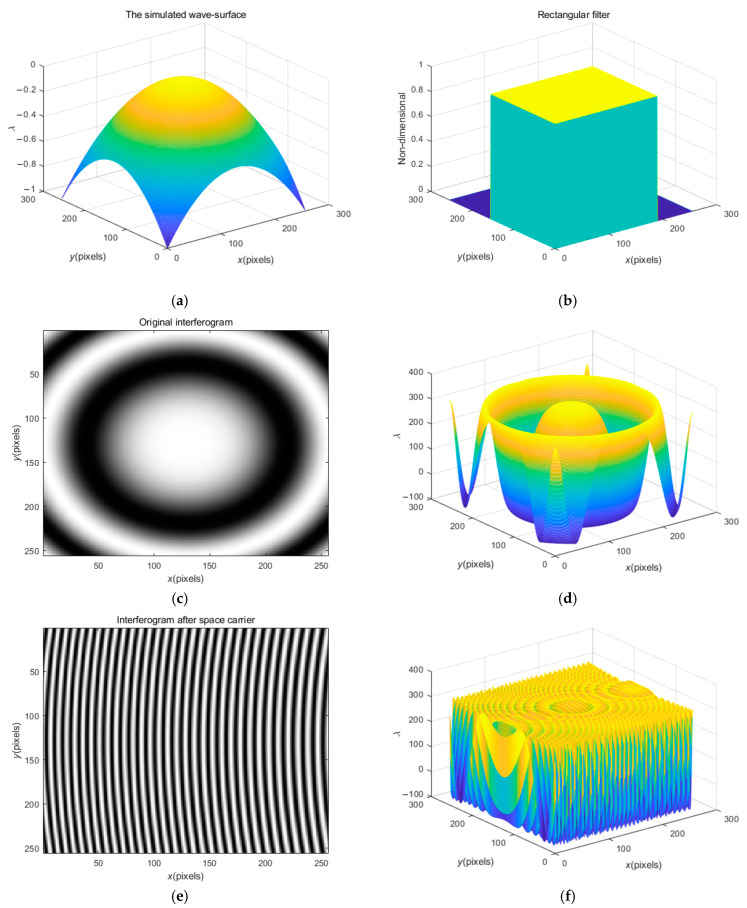

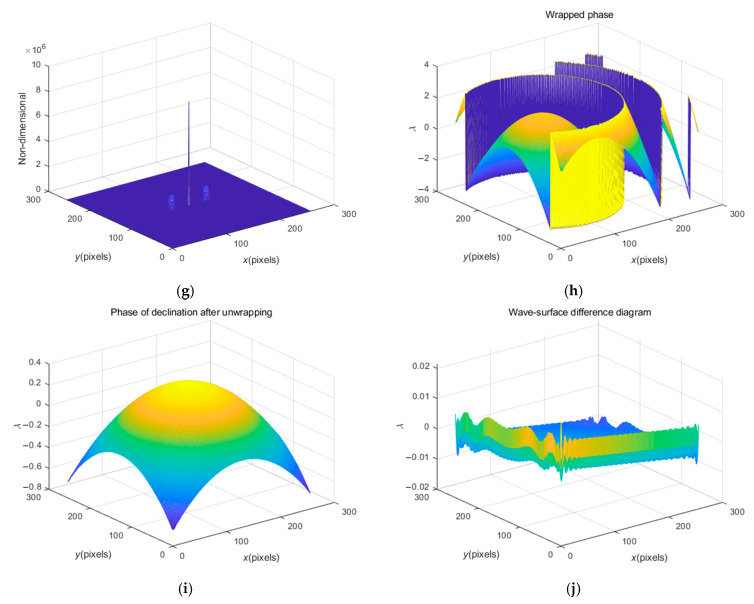
The simulated wave-surface and the rectangular filter, and the series of operation results based on rectangular filter when $f_{0}=32/256\;H$z: (**a**) the simulated wave-surface; (**b**) the rectangular filter; (**c**,**d**) the original interferogram displayed in the form of fringe pattern and stereogram; (**e**,**f**) the interferogram after carrier processing that is displayed in the form of fringe pattern and stereogram; (**g**) the spectrums of fundamental wave and the background spectrum; (**h**) the wrapped phase obtained; (**i**) the phase of declination after unwrapping; (**j**) the wave-surface difference diagram.

Let us first examine the series of operation results based on the rectangular filter when $f_{0}=32/256\;H$z. Figure 4a,b show the simulated wave-surface and the rectangular filter. Figure 4c,d show the original interferogram displayed in the form of a fringe pattern and stereogram. It is obvious that in the spectrum of this annular fringe pattern, the fundamental frequency and the background spectrum are in an overlapping state, and cannot be separated, so the FT method will fail, which is precisely the purpose of carrier processing. Figure 4e,f show the interferogram after carrier processing that is displayed in the form of a fringe pattern and stereogram. The fringes of this interferogram are basically linear, and the fundamental frequency and the background spectrum can be completely separated, which is suitable for subsequent spectral filtering operations. Figure 4g clearly shows the spectrums of fundamental wave and the background structure that can be separated. Filter Figure 4g to separate the spectrum of the first harmonic, and then carry out inverse FT and obtain the arctangent function as depicted in Equation (4), to further obtain the wrapped phase as demonstrated in Figure 4h that is wrapped in the principal value range $\left(-\pi ,\;\pi \right]$. To obtain the practical phase information, the appropriate number of integer multiples of 2π should be added at each pixel of the wrapped phase, which is one of the classical problems in optical signal processing, and is referred to as phase ‘unwrapping’, mentioned above [39].

Hence, Figure 4h needs to be further unwrapped, and the tilt effect caused by the carrier frequency can be eliminated, and then the measured phase can be recovered. The recovered phase is shown in Figure 4i. Figure 4j shows the wave-surface difference (error) diagram, which is the difference between the original simulated wave-surface and the recovered wave-surface, and it also contains the PVV $W_{\mathrm{pv}}=0.0342\left(\lambda \right)$ and RMSV $W_{\mathrm{rms}}=0.0033\left(\lambda \right)$ after solution. Since the overall error is between plus and minus 0.01 λ, the processing process meets the accuracy requirements.

**Figure 5:**
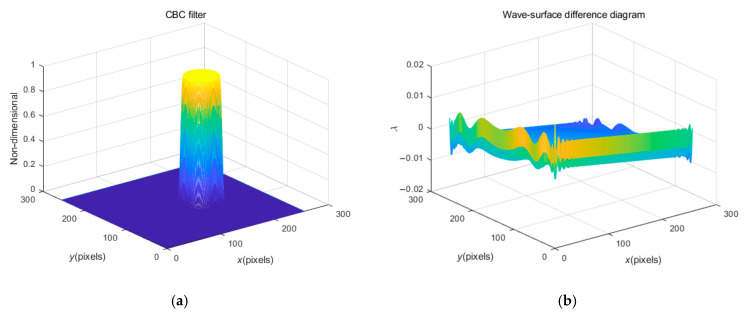
(**a**) The CBC filter; (**b**) the wave-surface difference (error) diagram, when $f_{0}=32/256\;H$z.

Figure 5 shows the CBC filter (a) and the wave-surface difference (error) diagram (b) when $f_{0}=32/256\;H$z. Compared with Figure 4j, the wavefront and boundary in Figure 5b appear smoother, reflecting less high-frequency noise, which improves the processing accuracy compared with the original simulated wavefront. It also contains the better PVV with $W_{\mathrm{pv}}=0.0320\left(\lambda \right)$.

**Figure 6:**
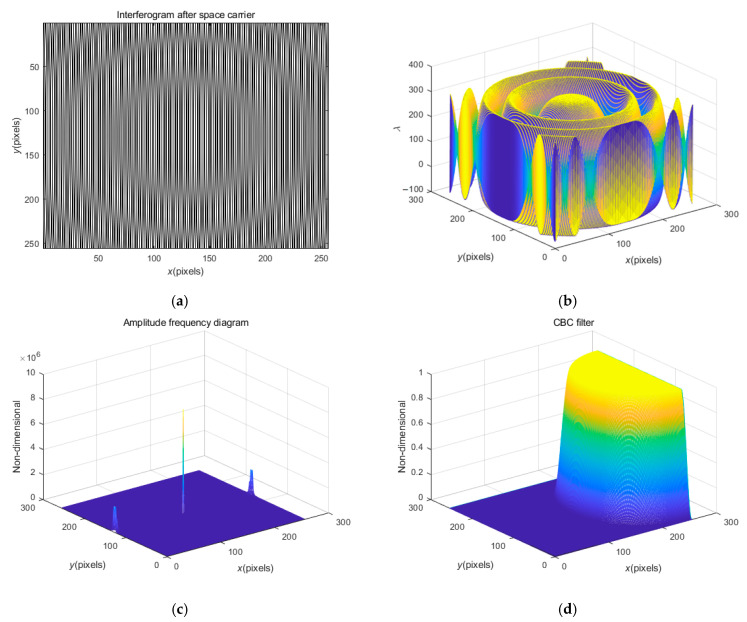
The series of operation results based on CBC filter when $f_{0}=128/256\;H$z: (**a**,**b**) the interferogram after carrier processing that is displayed in the form of fringe pattern and stereogram; (**c**) the split and truncated spectrogram; (**d**) the half CBC filter.

In Figure 2, a higher carrier frequency is not shown. As mentioned earlier, at a higher carrier frequency, such as $f_{0}=128/256$ Hz, the spectrum filtering method will fail. Figure 6 shows the series of operation results based on the CBC filter when $f_{0}=128/256\;H$z. Figure 6a is the interferogram after the space carrier, which clearly shows a newly generated Newtonian ring compound fringe that is clearly expressed in the stereogram of Figure 6b. Figure 6c shows the spectrum diagram. The frequency spectrum of the first harmonic is close to the boundary and has been cut off, which not only relates to the Newton ring but also causes the subsequent spectrum filtering distortion. Figure 6d shows half a filter, which also means that spectrum filtering cannot be completed. Therefore, the high carrier frequency not only represents the complexity of the carrier process and hardware operation, but also makes the whole algorithm useless. This is also the reason why the medium carrier frequency is recommended in practical operation.

## 3. Real-Time Detection

In this design, the practical test object is a precision circular flat crystal with a diameter of 100 mm (or 150 mm), and the signal acquisition is limited to the range of 256×256 pixels (or 426 × 426 pixels). In the practical measurement, the FPs collected by the CCD image sensor are circular or annular. As the FFT algorithm requires that the FP region must be rectangular to avoid the edge effect of the processing results, the extrapolation and interpolation techniques of interferograms are necessary [26]. Its essence is to extend the fringe pattern in space, extract the phase of the extended image, and then extract the effective area phase [40]. In this design, a bidirectional extrapolation and interpolation (BEI) technology is adopted. After extrapolation or interpolation, the interferogram can be processed with the FFT method described above to obtain the recovered phase. In the present study, the restored phase is compared with the processing results of a ZYGO interferometer, which verifies the effectiveness of the BEI technology and FFT method in interferogram processing.

### 3.1. The BEI Technology

**Figure 7:**
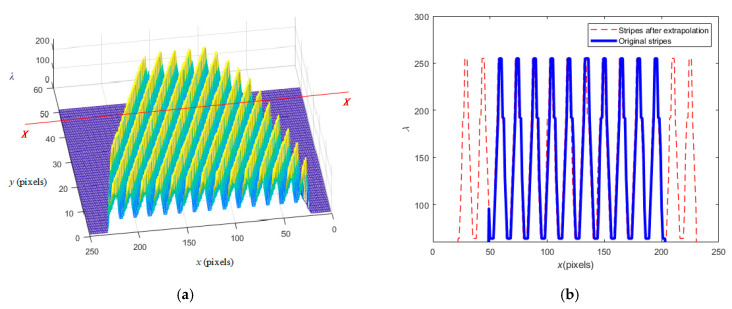
(**a**) The local stereogram of a circular interferogram; (**b**) the schematic diagram of extrapolation.

Figure 7a shows the local stereogram of a circular interferogram. The horizontal plane has two directions, one is the x direction (i.e., *X*–*X* direction), and the other is the vertical y direction (i.e., the implied *Y*–*Y* direction). Figure 7b is the sectional view along the *X*–*X* direction. The blue stripes here represent the existing actual stripes. The red stripes on both sides represent the stripes after extrapolation along the x direction. The extrapolation method starts from the extreme point of the edge of the blue stripe, takes the average period of the blue stripes as the period of the extrapolation stripes, and extends to the two outer sides with a sine curve. The intensity of the new interferogram obtained after extrapolation can be expressed as $i_{X}\left(x,\;y\right)$. 

The so-called bidirectional extrapolation refers to another extrapolation along the y direction to obtain another intensity interferogram $i_{Y}\left(x,\;y\right)$, and then the final extrapolation result image can be expressed by $i_{\mathrm{new}}\left(x,\;y\right)=\left[i_{X}\left(x,\;y\right)+i_{Y}\left(x,\;y\right)\right]/2$. The advantage of BEI technology is that it can not only fully consider the influence of carrier frequency $f_{0x}$ in the x direction, but also fully reflect the influence of carrier frequency $f_{0y}$ in the y direction. Therefore, compared with single-direction extrapolation, its accuracy can be obviously, effectively improved.

### 3.2. Testing and Cases

#### 3.2.1. Comparison of Test Results with ZYGO Interferometer

**Figure 8:**
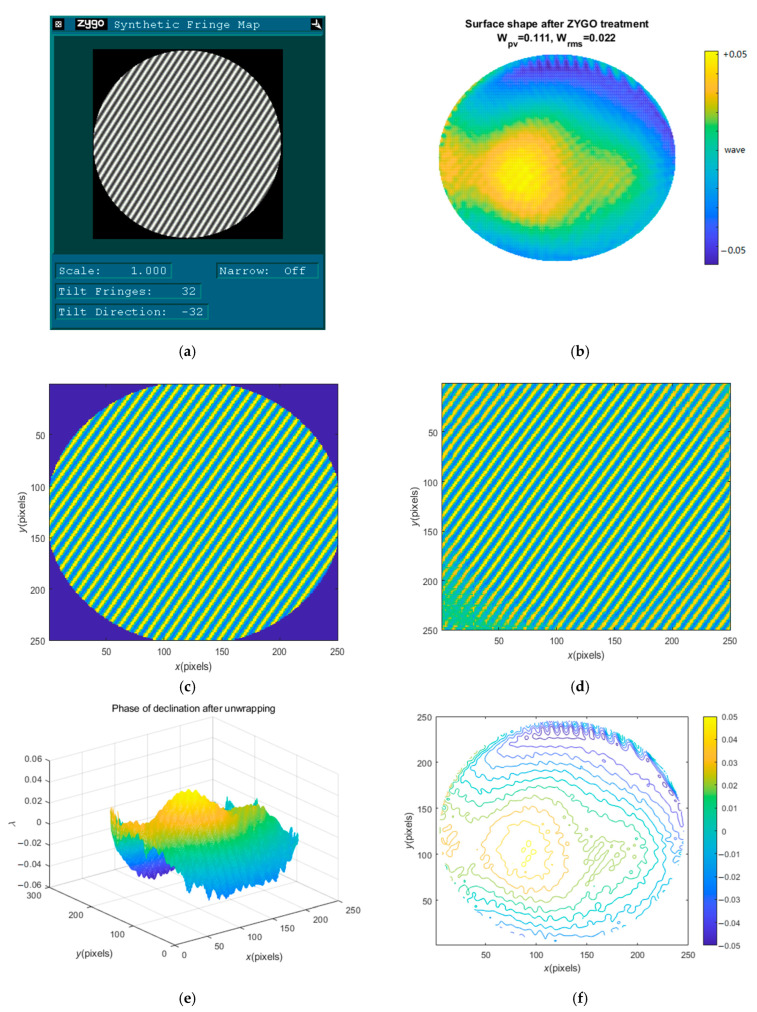
Comparison of Test Results with ZYGO Interferometer: (**a**) the interferogram collected when the surface of No. 1 workpiece is measured by ZYGO interferometer; (**b**) the recovery phase after ZYGO processing; (**c**) the interferogram used in this design; (**d**) interferogram after extrapolation; (**e**) the wave-surface after recovery; (**f**) the contour map of the recovery phase.

Figure 8a shows the interferogram collected when the surface of a workpiece (No. 1) is measured by a ZYGO interferometer. Figure 8b shows the recovery phase after ZYGO processing with $W_{\mathrm{pv}}=0.111\left(\lambda \right)$, $W_{\mathrm{rms}}=0.022\left(\lambda \right)$.

In order to effectively compare algorithms, the same carrier frequency and the same fringe tilt angle are used in this design, and the obtained interferogram is shown in Figure 8c. Figure 8d shows the FPs after extrapolation. Figure 8e shows the recovered phase, i.e., the phase of declination after unwrapping, with the implied values $W_{\mathrm{pv}}=0.1117\left(\lambda \right)$, $W_{\mathrm{rms}}=0.0217\left(\lambda \right)$. Figure 8f shows the contour map of the recovery phase. Compared with the result of Figure 8b, Figure 8e,f show considerable consistency, as well as the surface accuracy quality indexes, which also prove the effectiveness of the proposed algorithm and the adopted technology in the present study.

#### 3.2.2. Processing of Seriously Polluted Noise Images

**Figure 9:**
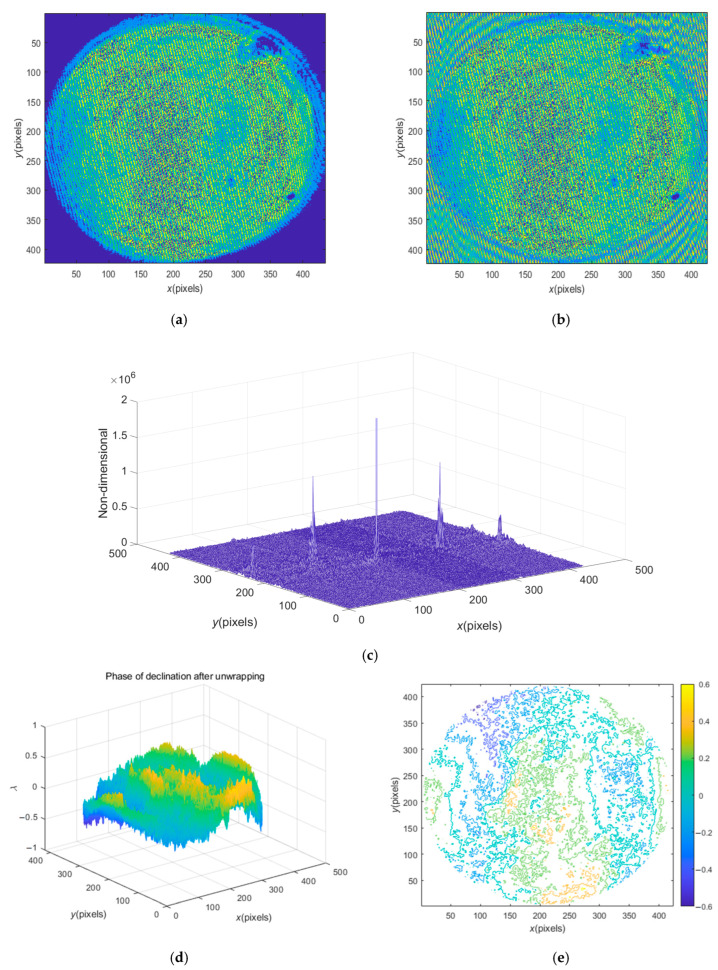
Processing of seriously polluted noise image of No. 2 workpiece: (**a**) the noise image; (**b**) the interferogram after extrapolation; (**c**) the spectrum diagram after extrapolation; (**d**) the restored surface shape with complex high-frequency noise signals; (**e**) the contour map of the recovery phase.

In order to test the wider applicability of the method proposed in this study, a heavily polluted noise interferogram of No. 2 workpiece is processed, as shown in Figure 9a. Figure 9b shows the interferogram after extrapolation, which approximately depicts the carrier effect in the surrounding area. Figure 9c is the spectrum diagram of Figure 9b. In the spectrum diagram, it can be seen that the high-frequency noise is very serious, covering the entire frequency domain. The spectrum diagram shows not only the spectrum of the first harmonic, but also the spectrum of the second harmonic. The spectrum amplitude of the second harmonic is also relatively large, so it cannot be ignored, which is another reason why a complex noise image is difficult to process. Figure 9d shows the restored surface shape with complex high-frequency noise signals, which need to be further filtered out by noise filtering technology in subsequent processing (not mentioned here). Figure 9e is the corresponding contour map, which vividly shows the noise pollution areas of different sizes.

#### 3.2.3. Processing of Annular Interferogram

**Figure 10:**
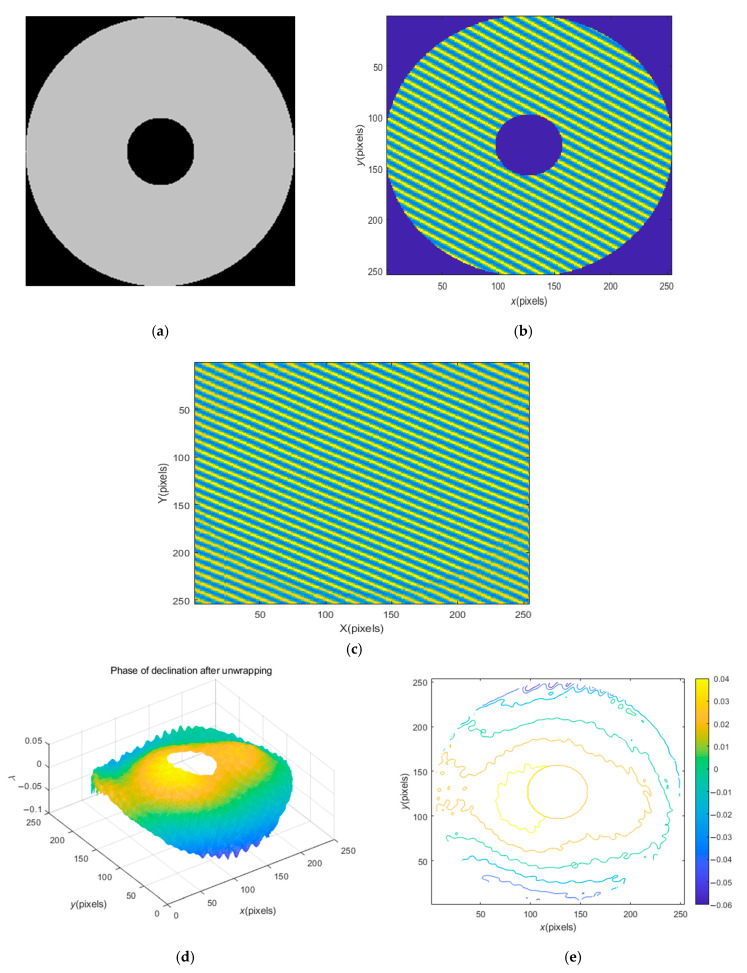
Processing of annular interferogram: (**a**) the annular No. 3 workpiece; (**b**) the annular interference diagram; (**c**) the interferogram after extrapolation and interpolation; (**d**) the recovery phase; (**e**) the contour map of the recovery phase.

Figure 10 shows the processing of annular No. 3 workpiece. Figure 10b is the interference diagram of workpiece (a). The above BEI extrapolation technology can also be used for interpolation. The interferogram after extrapolation and interpolation is shown in Figure 10c. Compared with extrapolation, the interpolation effect is better, and almost no trace of interpolation can be seen, which also reflects the robustness of the proposed BEI technology. Figure 10d,e show the recovered phase and contour map, which clearly describes the position and size of the central hole, as well as the surrounding surface fluctuations, and also verifies the effectiveness of the method proposed in the present study.

It should be emphasized that the image acquisition system is from the company Daheng Imaging [41]. Daheng Imaging provides users with various models and brands of image acquisition cards. The digital image acquisition card (based on the CameraLink interface) is a hardware device that converts the image signal of an analog camera through A/D conversion, or transfers the output signal of a digital camera (type: DH-51) to computer memory or video memory through the computer bus, so that the computer can process, store, and display the field image captured by the camera in real time. The digital image acquisition card (type: CG-51) collects the digital signal to the PC losslessly through the digital interface in the form of digital to digital based on the camera software DH51_Windows_CN-EN_32/64bits and the acquisition card software CG_VT_CN_user _cd_x32/64. The algorithm of the optical process is implemented in MATLAB (R2011b, MathWorks, Natick, MA, USA), which automatically calls the image obtained by the digital acquisition card, and then carries out independent processing.

## 4. Conclusions

Real-time detection application of a laser interference sensing measurement system based on a 4R manipulator system is designed and investigated for the purpose of real-time online detection in the production process and for the requirement of rapid processing for the interferogram. Some concluding remarks can be drawn from the results:

- A 4R mobile manipulator (MM) system has been designed and integrated with the laser ISM system, which is a structural fusion method and has achieved the purpose of real-time online detection. The 4R MM system and the ISM system were designed independently and combined by specific connectors. This has laid the foundation for their respective upgrading and performance improvement, which provides an effective idea for similar laser test projects;
- The existing commercial interferometers are of high accuracy, but most of them are still not suitable for real-time measurement of online machining workpieces in the workshop. Instead, the workpiece needs to be transferred to a specific laboratory to realize the test. Therefore, this testing system was built to achieve real-time measurement in the workshop. Although this design aims at 3D detection, it focuses more on the detection of precise part surfaces, which is actually closer to the measurement of precise flatness of precise part surfaces.

## Figures and Tables

**Table 1:** The detailed PVVs and RMSVs under different carrier frequencies, $f_{0}=2/256,\;4/256,\;8/256,\;16/256,\;32/256,\;64/256,128/256\;\mathrm{Hz}$, respectively.

Title 1	*f*_0_ = 2/256	*f*_0_ = 4/256	*f*_0_ = 8/256	*f*_0_ = 16/256	*f*_0_ = 32/256	*f*_0_ = 64/256	*f*_0_ = 128/256
Rec_PVV ^1^	0.8864	0.1368	0.0830	0.0525	0.0342	0.0244	invalid
Rec_RMSV	0.1403	0.0167	0.0062	0.0038	0.0033	0.0032	invalid
CBC_PVV	0.9579	0.1647	0.0830	0.0490	0.0320	0.0241	invalid
CBC_ RMSV	0.1325	0.0191	0.0061	0.0037	0.0033	0.0032	invalid

^1^ ‘Rec’ refers to rectangular filter.

## Data Availability

Not applicable.

## Nomenclature

FPP	Fringe Projection Profilometry
SPI	Speckle Pattern Interferometry
ISM	Laser Interferometric Sensing Measurement
MM	Mobile Manipulator
CCD	Charge Coupled Device
FT	Fourier Transform
FFT	Fast Fourier Transform
CBC	Cosine Banded Cylindrical
BEI	Bidirectional Extrapolation and Interpolation
PST	Phase Shifting Technique
SCPS	Space Carrier Phase Shift
FPs	Fringe Patterns
PVV	Peak and Valley Value
RMSV	Root-Mean-Square Value
OPD	Optical Path Difference
PZT	A Type of Piezoelectric Ceramic Actuator
ZYGO	A Type of Interferometer
